# Reproductive aspirations, contraception use and dual protection among adolescent girls and young women: the effect of motherhood and HIV status

**DOI:** 10.1002/jia2.25558

**Published:** 2020-08-31

**Authors:** Elona Toska, Lucie Cluver, Christina A Laurenzi, Camille Wittesaele, Lorraine Sherr, Siyanai Zhou, Nontokozo Langwenya

**Affiliations:** ^1^ Centre for Social Science Research University of Cape Town Cape Town South Africa; ^2^ Department of Sociology University of Cape Town Cape Town South Africa; ^3^ Department of Social Policy and Intervention University of Oxford Oxford United Kingdom; ^4^ Department of Child and Adolescent Psychiatry University of Cape Town Cape Town South Africa; ^5^ Department of Global Health Institute for Life Course Health Research Stellenbosch University Cape Town South Africa; ^6^ Department of Infectious Disease Epidemiology Faculty of Epidemiology and Population Health London School of Hygiene & Tropical Medicine London United Kingdom; ^7^ Institute for Global Health University College London London United Kingdom; ^8^ Department of Statistics University of Cape Town Cape Town South Africa; ^9^ Oxford Research South Africa East London South Africa

**Keywords:** adolescents, motherhood, HIV, contraception, dual protection, South Africa

## Abstract

**Introduction:**

There is a growing interest in adolescent motherhood and HIV among policymakers and programme implementers. To better shape services and health outcomes, we need evidence on reproductive aspirations and contraception use in this high‐risk group, including the effect of motherhood and HIV status. We report data from a large survey of adolescent girls and young women conducted in a mixed rural‐urban district in South Africa.

**Methods:**

Quantitative interviews were conducted with 1712 adolescent girls and young women (ages 10 to 24): 336 adolescent mothers living with HIV (AMLHIV), 454 nulliparous adolescent girls living with HIV (ALHIV), 744 HIV‐negative adolescent mothers (control adolescent mothers) and 178 HIV‐negative nulliparous adolescent girls (nulliparous controls) in 2018 to 2019. Standardized questionnaires included socio‐demographic measures, reproductive health and contraception experiences. Reproductive aspirations were measured as the number of children participants wanted to have. Dual protection was computed as use of both hormonal and barrier contraception or abstinence. Multivariate logistic regression and marginal effects models in STATA 15 were used to test associations between HIV status, adolescent motherhood and outcomes of reproductive aspirations, contraception use and dual protection, controlling for covariates.

**Results and discussion:**

Nearly 95% of first pregnancies were unintended. Over two‐thirds of all participants wanted two or more children. Hormonal contraception, condom use and dual protection were low across all groups. In multivariate regression modelling, ALHIV were less likely to report hormonal contraception use (aOR 0.55 95% CI 0.43 to 0.70 *p* ≤ 0.001). In marginal effects modelling, adolescent mothers – independent of HIV status – were least likely to report condom use at last sex. Despite higher probabilities of using hormonal contraception, rates of dual protection were low: 17.1% among control adolescent mothers and 12.4% among AMLHIV. Adolescent mothers had the highest probabilities of not using any contraceptive method: 29.0% among control mothers and 23.5% among AMLHIV.

**Conclusions:**

Among adolescent girls and young women in HIV‐endemic communities, reproductive aspirations and contraceptive practices affect HIV risk and infection. Tailored adolescent‐responsive health services could help young women plan their pregnancies for when they are healthy and well‐supported, and help interrupt the cycle of HIV transmission by supporting them to practice dual protection.

## 1 INTRODUCTION

More than 50% of all births in sub‐Saharan Africa are to 15‐to‐19‐year‐old women [1]. By 2030, there will be nearly 1.5 million adolescent and young mothers living with HIV worldwide [2]. The majority of these pregnancies are unintended, and may result from violent and inequitable relationships [3, 4, 5, 6]. Despite these difficult first pregnancies, qualitative research from sub‐Saharan Africa suggests that adolescent girls and young women have strong reproductive aspirations, regardless of their HIV status. To date, no quantitative research has examined the effect of HIV status and motherhood on reproductive aspirations and practices.

The children of adolescent mothers have higher risks of preterm birth, low birthweight and mortality [7, 8, 9, 10]. Adolescent mothers are less likely to access contraception services and use postpartum contraception [11]. Nearly half of all unintended pregnancies among adolescent mothers end with terminations of pregnancy, most of which are unsafe [12, 13]. Moreover, in several sub‐Saharan African countries, many adolescent girls and young women report more than one pregnancy before age 20 [14], including adolescent girls living with HIV (ALHIV) [10, 15, 16]. Rapid repeat pregnancies within adolescence may pose further biological, developmental and economic risks for both the mother and her children.

The risks facing adolescent mothers are amplified for those living with HIV, who face additional vulnerabilities [17, 18, 19]. Adolescents living with HIV who become pregnant experience less consistent engagement in antenatal and HIV care, intermittent viral suppression [20, 21, 22], delayed initiation of antiretroviral therapy (ART) [23] and poorer access to infant HIV testing [24, 25, 26]. Consequently, adolescent mothers living with HIV (AMLHIV) in sub‐Saharan Africa are likelier than older mothers with HIV to transmit HIV to their children [26], despite global reductions in vertical HIV transmission [27].

More evidence about the syndemic of adolescent pregnancy and HIV is needed to inform future programming [18]. We present initial findings from a large study of adolescent mothers living with and without HIV, with comparison groups of nulliparous adolescent girls (N = 1712). This study addresses two important questions: First, how do reproductive aspirations, contraception use and dual protection practices of adolescent girls and young women vary by motherhood? Second, do experiences differ by adolescent mothers’ HIV status?

## 2 METHODS

The study was conducted in a mixed urban‐rural health district of the Eastern Cape Province, South Africa. We interviewed n = 1712 adolescent girls and young women between March 2018 to July 2019, of whom n = 1027 had delivered their first child before age 20. We utilized six parallel sampling strategies to reach adolescent mothers (independent of their HIV status), alongside nulliparous adolescent girls with matched demographic profiles. An advisory group of adolescent mothers co‐developed these recruitment methods with the research team for hard‐to‐reach adolescent mothers. For each recruitment channel, we recorded refusals and consenting adolescents. First, we included all district health facilities (n = 73). In health facilities, we searched all patient files of adolescent girls (10 to 19 years) who had ever initiated HIV treatment (whether still in HIV care or not) and interviewed them at home – 97% enrolled. Second, we used case files at all maternity obstetric units (n = 9) to identify all adolescent mothers (including AMLHIV) who were contacted through nurses, community healthcare workers in person or over the phone – 95% enrolled. Third, we randomly selected district secondary schools (n = 43) and interviewed adolescent girls who had recently given birth or dropped out of school due to pregnancy or motherhood – 98% enrolled. Fourth, we interviewed neighbouring adolescent girls of those approached through clinic files, which reduced any unintended stigmatization and provided a demographically matched control group. Fifth, we used referrals by social workers and NGO service providers to identify adolescent mothers who may be especially vulnerable – all eligible enrolled in the study (n = 95). Lastly, we included community referrals by adolescent mothers themselves – important in contexts where many adolescents do not access any services – all those eligible enrolled (n = 51).

Voluntary informed consent was obtained from adolescents and their caregivers when adolescents were under 18, following international and national guidelines for consent among vulnerable populations. Data collection tools were piloted with n = 25 ALHIV and nine adolescent mothers. Ethical approvals were obtained from the Universities of Oxford (R48876/RE001,SSD/CUREC2/12–21) and Cape Town (HREC226/2017,CSSR2013/4), Eastern Cape Departments of Health and Basic Education and participating health and educational facilities. Participants did not receive financial remuneration, but were awarded a participation certificate and small gift pack selected by our Teen Advisory Group, including toiletries for adolescents and infants.

We used measures and scales validated in South Africa, where possible. *Socio‐demographic* items (age, residence type (rural/urban), housing (formal/informal), household poverty and food insecurity) have been reported in detail elsewhere [19, 28]. *Age at first parity* was measured through self‐report and validated with oldest child age from children’s medical records. *Unintended first pregnancy* was measured by assessing whether the pregnancy resulting in the first child was unwanted or unplanned. *Reproductive aspiration* was measured by asking how many children participants desire and was dichotomized for analyses (1 = want 2 or more children; 0 = wants none or 1 child). *Hormonal contraception* was computed from three variables: current self‐reported use of oral contraceptives, injectables or implant: participants reporting using any form was coded as 1. *Condom use at last sex* was defined as using a condom for the entire duration of the most recent sexual act. *Dual protection* was computed by combining adolescents reporting both hormonal contraception and condom use at last sex. Abstinent adolescents were marked as dually protected. Participants were coded as having *no protection* if they reported no hormonal contraception nor condom use, and were not abstinent. *Current ART use* was defined as self‐reported ART use during the interview. Relationship factors included: *relationship status* (yes/no), *partner type* (boyfriend/girlfriend/husband/wife vs. casual), *partner HIV status knowledge* (unknown, HIV negative, HIV positive), all self‐reported by participants.

All analyses were conducted using STATA 15. First, socio‐demographic characteristics, reproductive aspirations, contraception and dual protection frequencies were computed for the full sample and for four sub‐groups by HIV status and motherhood: (1) *AMLHIV*, (2) adolescent girls and young women living with HIV who have not initiated childbearing (*nulliparous ALHIV*), (3) HIV‐negative adolescent mothers (*control adolescent mothers*), and (4) HIV‐negative adolescent girls and young women who have not initiated childbearing (*nulliparous controls*). Second, pairwise correlations among socio‐demographics and relationship variables were computed to check collinearity and to determine which factors were included in additional analyses. Third, associations between HIV status and motherhood on outcomes were explored using multivariate logistic regression models, controlling for socio‐demographic variables that were significantly different across four sub‐groups. *p*‐values were adjusted for multiple outcome testing using the Benjamini–Hochberg approach [29]. Fourth, predicted probabilities of reporting each outcome for the four sub‐groups were computed, holding all included socio‐demographic factors at their mean values.

## 3 RESULTS AND DISCUSSION

### 3.1 Participant characteristics

Table 1 presents frequencies of socio‐demographic characteristics and reproductive aspirations and experiences for the full sample (N = 1712) and four sub‐groups defined above: (i) AMLHIV (n = 336, 20%), (ii) nulliparous ALHIV (n = 454, 27%), (iii) control adolescent mothers (n = 734, 44%) and (iv) nulliparous controls (n = 188, 10%). Participants’ mean age was 17.6 years (IQR 16 to 19 years, SD = 2.6), and average age at first child among n = 1045 mothers was 16.5 (SD 1.8). AMLHIV, on average, had their first child slightly later at 17.3 years (SD 2.2, *p* ≤ 0.001). One‐quarter of participants lived in rural areas. AMLHIV were more likely to live in informal housing (*p* ≤ 0.001). One‐quarter of participants reported past‐week food insecurity. The study’s participants live in challenging socio‐economic environments, reflecting the living situations of most adolescents in the region.

**Table 1:** Socio‐demographic and relationship characteristics of adolescent girls and young women by HIV and motherhood

Characteristics	AMLHIV (n = 336)	Nulliparous ALHIV (n = 454)	Control adolescent mothers (n = 744)	Nulliparous controls (n = 178)	All participants (n = 1712)	*p*‐value
Age (mean, SD)	19.8 (1.9)	16.3 (2.9)	17.8 (1.5)	16.3 (3.0)	17.6 (2.6)	≤0.001
Age at first child (mean, SD)^a^	17.3 (2.2)		16.3 (1.6)		16.5 (1.8)	≤0.001
Rural residence (n, %)	86 (25.4)	109 (24.3)	223 (30.0)	47 (26.4)	465 (27.2)	0.141
Informal housing (n, %)	80 (23.6)	63 (14.0)	155 (20.8)	15 (8.4)	313 (18.3)	≤0.001
Household poverty (n, %)	276 (81.4)	315 (69.8)	591 (79.4)	110 (61.8)	1292 (75.5)	≤0.001
Food insecurity (n, %)	99 (29.2)	98 (21.7)	195 (26.2)	43 (24.2)	435 (25.4)	0.102
Currently on ART (n, %)	291 (85.8)	409 (90.7)	NA	NA	700 (88.6)	0.034
Sexually active in the past year (N, %)	318 (93.8)	111 (24.6)	696 (93.6)	73 (41.0)	1198 (70.0)	≤0.001
In a relationship (n, %)	246 (74.1)	113 (25.2)	477 (64.5)	74 (41.8)	910 (53.6)	≤0.001
Partner HIV status (n, %)						≤0.001
Unknown	124 (49.0)	72 (62.6)	126 (26.2)	32 (42.7)	354 (38.3)	
HIV‐negative	62 (24.8)	35 (29.7)	352 (73.2)	43 (57.3)	492 (53.3)	
HIV‐positive	67 (26.7)	8 (6.8)	3 (0.6)	0 ()	78 (8.4)	
Last sexual partner casual (n, %)	87 (25.7)	337 (74.7)	268 (36.0)	106 (59.6)	798 (46.6)	≤0.001
Reproductive aspirations, contraception and dual protection						
Aspirations (mean, SD)	2.0 (0.8)	1.8 (0.9)	1.8 (0.8)	2.2 (1.7)	1.9 (1.0)	≤0.001
Aspirations – want 2 or more children (n, %)	252 (74.3)	303 (67.2)	480 (64.5)	145 (81.5)	1180 (68.9)	≤0.001
First child pregnancy unintended (n, %)^a^		274 (93.2)		716 (95.2)	990 (94.7)	0.193
Hormonal contraception (n, %)	214 (63.1)	81 (18.0)	474 (63.7)	55 (30.9)	824 (48.1)	≤0.001
Condom use at last sex (n, %)^b^	99 (31.1)	61 (55.0)	178 (25.6)	39 (53.4)	377 (31.5)	≤0.001
Dual protection (n, %)	72 (21.2)	46 (10.2)	136 (18.3)	32 (18.0)	286 (16.7)	≤0.001
No protection (n, %)	93 (27.4)	34 (7.5)	218 (29.3)	22 (12.4)	367 (21.4)	≤0.001

^a^ Data available for n = 1045 adolescent mothers only

^b^ Among sexually active participants only n = 1198.

AMLHIV, adolescent mothers living with HIV; SD, standard deviation; ART, antiretroviral therapy.

Nearly all adolescent mothers – independent of HIV status – had at least one sexual partner in the last year, with over half reporting being in a relationship. A third of all participants knew their partner’s HIV status, with three‐quarter of control adolescent moms reporting they knew this (almost all partners were reported as HIV negative).

On average, participants wanted to have two children (mean 1.9, IQR, SD = 1.0). Over two‐thirds of all participants wanted to have at least two children, though almost 95% of all first childbearing pregnancies were unintended. Just under half of the participants reported using hormonal contraception, 31.5% of sexually active participants reported condom use at last intercourse, and 16.7% reported using both condoms and hormonal contraception for dual protection. Twenty percent reported using no methods of contraception or HIV prevention at last sex.

### 3.2 Effect of HIV and motherhood on reproductive practices

**Figure 1:**
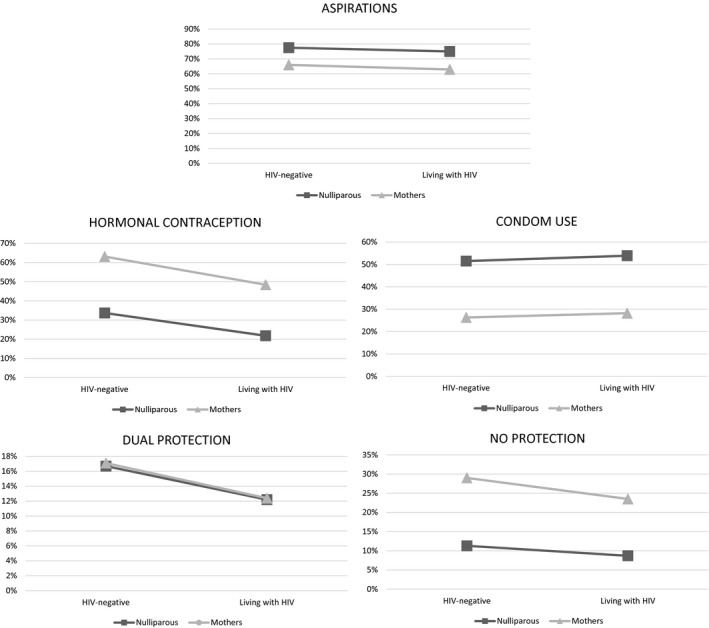
Predicted probabilities of reporting outcomes among adolescent girls and young women by HIV status (n = 1712).

Results of multivariate regressions for each of the five outcomes adjusted for socio‐demographic variables are included in Table 2, with predicted probabilities of reporting the outcomes in Figure 1. All the rates reported below are adjusted predicted probabilities of each outcome being reported, holding age, informal housing and poverty at their mean values. Independent of HIV status, adolescent mothers were less likely to report wanting >2 children (aOR 0.57, CI 0.43 to 0.75), possibly due to the challenges already experienced [30]. Predicted probabilities – adjusted to take into account age, informal housing and poverty – showed that aspirations for two or more children were lowest among AMLHIV (62.9%) and highest among nulliparous controls (77.4%).

**Table 2:** Multivariate regression models testing the effect of HIV and motherhood on reproductive aspirations, contraception and dual protection (n = 1712)

Variables included in each model	Outcome 1: reproductive aspirations (n = 1712)		Outcome 2: hormonal contraception (n = 1712)		Outcome 3: condom use at last sex (n = 1198)	
	aOR (95% CI)	*p*‐value	aOR (95% CI)	*p*‐value	aOR (95% CI)	*p*‐value
Age	1.13 (1.08 to 1.18)	≤0.001*	1.30 (1.24 to 1.37)	≤0.001*	1.11 (1.03 to 1.19)	0.006
Housing (informal)	1.56 (1.17 to 2.07)	0.002	1.23 (0.93 to 1.63)	0.147	1.57 (1.15 to 2.15)	0.004
Poverty (missing at least one basic necessity)	1.13 (0.89 to 1.44)	0.319	0.85 (0.66 to 1.11)	0.231	0.50 (0.37 to 0.68)	≤0.001*
HIV‐positive status	0.87 (0.69 to 1.11)	0.261	0.55 (0.43 to 0.70)	≤0.001*	1.10 (0.82 to 1.49)	0.515
Motherhood	0.56 (0.42 to 0.75)	≤0.001*	3.37 (2.58 to 4.40)	≤0.001*	0.34 (0.29 to 0.47)	≤0.001*

Variables included in each model	Outcome 4: dual protection at last sex (n = 1712)		Outcome 5: no protection at last sex (n = 1712)	
	aOR (95% CI)	*p*‐value	aOR (95% CI)	*p*‐value
Age	1.28 (1.20 to 1.36)	≤0.001*	1.07 (1.00 to 1.13)	0.037
Housing (informal)	1.53 (1.11 to 2.10)	0.009	0.68 (0.50 to 0.94)	0.021
Poverty (missing at least one basic necessity)	0.55 (0.41 to 0.74)	≤0.001*	1.73 (1.25 to 2.38)	0.001
HIV‐positive status	0.69 (0.51 to 0.93)	0.015	0.75 (0.57 to 1.00)	0.047
Motherhood	1.02 (0.74 to 1.43)	0.886	3.21 (2.25 to 4.58)	≤0.001

* Significant at 0.001 level, when adjusted using the Benjamini–Hochberg correction for multiple outcome testing.

Hormonal contraception showed differences by both adolescent motherhood and HIV status. Adolescent motherhood was associated with substantially higher rates of hormonal contraception use (aOR 3.37, 95% CI 2.58 to 4.40) compared to nulliparous girls. Amongst adolescent mothers, AMLHIV had a lower predicted probability of reporting hormonal contraceptive use (48.4% vs. 63.1% amongst control adolescent mothers). The opposite pattern was observed for condom use at last sex among sexually active participants (aOR 0.34, 95% CI 0.29 to 0.47), with higher rates amongst adolescents who had not yet had children: 52.5% among controls and 53.9% among ALHIV. Adolescent motherhood rates of condom use were slightly higher amongst ALMHIV (28.2%) than adolescent mother controls (26.3%). Dual protection, recommended for all adolescent girls and mothers in national guidelines, remained extremely low (16.7%). ALHIV who had not yet had children had lowest rates of dual protection (10.2%). Adolescent mothers were more likely to report no protection at last sex (aOR 3.21, 95% CI 2.25 to 4.58). The likelihood of reporting no protection was highest among control adolescent mothers (29.0%).

### 3.3 Implications for service provision and research

These findings highlight important paradigm shifts that are needed to provide effective health and social services to adolescents and young women. First, they highlight the importance of responding to adolescent girls’ parenthood aspirations and needs, not only their risk profiles. Adolescent girls and young women – regardless of HIV status – aspire to have families, with over two‐thirds of them wanting two or more children. If acknowledged in respectful and age‐appropriate ways, these aspirations provide an opportunity to engage adolescents in integrated sexual and reproductive health (SRH) services, including safe conception, into HIV care and treatment services. Rates of dual protection – protection from sexually transmitted infections (STIs) and access to family planning – were low among all participants, only slightly higher than the rates of dual protection documented among young women in two South African studies over a decade ago [31, 32]. Nulliparous ALHIV had the lowest rates of contraception and dual protection use. Instead of advising ALHIV to refrain from sex, relationships or related risk‐taking [33], providers should listen to the aspirations and life circumstances of young women to effectively support them to attain positive SRH outcomes – for themselves, their partners and their children [13].

Second, the timing of these pregnancies is critical. With nearly 95% reporting unintended first pregnancies, adolescent girls and young mothers – particularly ALHIV – must be supported to time their pregnancies for when they are wanted, ideally when women are healthy and well‐supported emotionally, socially and financially [34]. Improving adolescent maternal outcomes and fostering child development also relies on preventing rapid repeat pregnancies. Moreover, given high rates of intimate partner violence and power‐inequitable relationships reported by adolescent girls and young women, additional analyses integrating these complex considerations are needed [35]. Further research is needed on the experiences of AMLHIV across HIV, SRH and contraception and maternal and child health services, to better integrate and adapt them to the unique needs of this age group.

Third, access to and use of contraception, including consistent condom use, was low among participants. Motherhood provides an opportunity to support young women to initiate contraception, however, we found increased hormonal contraception seemed to occur in parallel with reduced condom use. Adolescent girls and young mothers – regardless of HIV status – must access a contraceptive methods mix that is consistent, accessible and appropriate for their life stage and the precarious environments they live in [13].

Fourth, the contraceptive and HIV prevention gap reported in this study is extremely time‐sensitive: the risk of new HIV infections among adolescent girls increases once they have had their first child in adolescence [36]. Existing research confirms the urgent need for combination prevention to reduce HIV and STI incidence among adolescent girls and young women, including dual protection [37, 38]. Low rates of initiation and retention on ART, especially in the postpartum period, combined with high rates of repeated pregnancies in this age group [11], make AMLHIV highly vulnerable to passing HIV on to their children.

Finally, this analyses does not include data on ART access and use alongside conventional dual protection methods. Attaining and maintaining viral suppression – alongside an age and life‐stage appropriate contraceptive mix – is central to reducing onward HIV transmission rates. While timely viral load information is not readily available in low resource settings, future analyses on this topic is critical.

This study has several limitations. First, all outcomes are self‐reported, and may underestimate actual experiences of adolescent girls and young women. Data on ART access and viral suppression were not yet available for these analyses. Differential reporting by age and HIV status may have affected results, given potential increased stigma attached to younger motherhood and living with HIV. Second, the data are from South Africa and may not be generalizable. Nonetheless, we conducted the study in a resource‐constrained setting, comparable to others in Southern Africa, which may allow for cross‐cutting lessons. Third, cross‐sectional data limits our ability to draw causal inferences. More complex analyses to investigate potential interactions between HIV status, motherhood and relationship/partner factors, including longitudinal follow‐up, are needed to understand factors shaping the SRH practices of adolescent girls and young women. Fourth, as the sample was young (average age 16.2 years), the study most likely underestimates rates of adolescent pregnancy. Finally, this short report includes limited information on the sexual partners of adolescent girls and young women, which is not yet available in this data. Future research on partners will be important in understanding the dynamics of early pregnancy, reproductive aspirations and practices for these girls and young women.

Despite the above limitations, this study has several strengths. First, it is the first and largest quantitative analyses of reproductive aspirations and practices of adolescent girls and young mothers living in HIV‐endemic communities. Second, given the high levels of stigmatization reported by ALHIV [39, 40] and adolescent mothers [30], conventional recruitment techniques for this sample would have resulted in a biased sample. The research team designed a systematic sampling approach prioritizing non‐stigmatization and use of community and peer networks to increase reach and uptake among this group.

## 4 CONCLUSIONS

As new infections among adolescents persist, and as more ALHIV reach childbearing age [5], we need to better understand how to engage adolescent mothers in comprehensive, tailored health services to effectively reduce HIV‐related morbidity and mortality [17]. Safe conception considerations – planned pregnancies that coincide with viral suppression, but also socio‐emotional readiness – must be integrated in HIV and SRH service provision for adolescent girls and young women living with HIV. It is also critical to shift away from a risk‐centred narrative, promoting more nuanced evidence on young women’s sexual and reproductive health needs and practices, especially among ALHIV [15, 30, 41]. Additional research is needed to understand which factors support adolescent girls and young women to use dual protection and have well‐timed, supported pregnancies, particularly in the context of HIV. Our preliminary results echo calls for the integration of HIV and SRH services made at recent academic, policy and donor forums – the time for saving future generations is now!

## COMPETING INTERESTS

Study sponsors were not involved in study design, data collection, analyses nor interpretation, the writing of this manuscript, nor the decision to submission of this manuscript. ET wrote the first draft and no honorarium, grant or other form of payment were given to produce the manuscript.

## AUTHOR’S CONTRIBUTIONS

ET, LC, LS and CW designed and implemented the overall study. ET and LC conceptualized the analyses. ET led the analyses, with support from LC, NL and SZ. ET, LC and CL wrote the manuscript’s first draft. ET and CL led on the revisions following peer reviews. All authors provided edits and feedback on manuscript content and have approved the final draft.

## ABBREVIATIONS

AIDS, acquired immunodeficiency syndrome; ALHIV, adolescent girls living with HIV; AMLHIV, adolescent mothers living with HIV; ART, antiretroviral therapy; HIV, human immunodeficiency virus; SRH, sexual and reproductive health; STIs, sexually transmitted infections.
